# Global Research Priorities for Understanding and Improving Respectful Care for Newborns: A Modified Delphi Study

**DOI:** 10.9745/GHSP-D-21-00292

**Published:** 2022-02-28

**Authors:** Hagar Palgi Hacker, Elena Ateva, R. Rima Jolivet, Bushra Al-makaleh, Theresa Shaver, Emma Sacks

**Affiliations:** a George Washington University, Washington, DC, USA.; b White Ribbon Alliance, Washington, DC, USA.; c Women & Health Initiative, Harvard T.H. Chan School of Public Health, Boston, MA, USA.; d Independent public health advisor, Sanaa, Yemen.; e U.S. Agency for International Development Contractor Social Solutions International, Inc., Washington, DC, USA.; f Department of International Health, Johns Hopkins Bloomberg School of Public Health, Baltimore, MD, USA.

## Abstract

**Introduction:**

As the respectful maternity care agenda expands, it is necessary to understand what the priorities are for improving respectful, dignified, and high-quality care for newborns. To catalyze and inform a developing research field, we undertook a prioritization exercise to identify global research questions.

**Methods:**

We used a modified Delphi process to identify potential research priorities for understanding and addressing respectful care for newborns. Based on a literature review and expert discussions, we sent an open-ended questionnaire to participants in Round 1. The results were consolidated and used to create the Round 2 questionnaire, which asked participants to rank the preliminary list of research questions in 3 categories (descriptive, implementation, and measurement). Responses were weighted by rank and collated to generate a prioritized, consensus-based list of research questions.

**Findings:**

Round 1 resulted in 70 research questions from 25 respondents, and 52 participants completed the Round 2 ranked survey. Most participants identified themselves as researchers (53.2%), program designers, implementers, or evaluators (56.5%). More than half of the participants reported working primarily in low-income countries (66%). Top descriptive questions were on understanding manifestations and definitions of disrespectful care among newborns and which perceptions and beliefs held by health workers affect the quality of care provided. Top implementation questions were around how to promote respectful care as a standard, challenges faced by health facilities, and identification of effective advocacy strategies. Top measurement questions were on quantitative and qualitative metrics and the impact of experiences on health outcomes.

**Conclusions:**

This study developed, for the first time, a prioritized list of research questions focusing exclusively on respectful care for newborns. The study highlighted the absence of agreed-upon terminology and tools needed to advance both theoretical and practical efforts. This list should guide researchers and other stakeholders in developing further research.

## Abstract (precis)

To inform the developing research field of respectful care, we identified global research questions that are specific to respectful newborn care. The top descriptive, implementation, and measurement questions focused primarily on defining, promoting, measuring, and advocating for respectful care.

## Teaser

**Key Findings:**

- We undertook a modified Delphi exercise to learn what experts think are the key questions regarding respectful care for newborns, resulting in the first prioritized list of research questions that focus on respectful care for newborns.
- This study is the first to offer a working definition of respectful care for newborns and develop a ranked list of research questions focusing exclusively on respectful care for newborns.

**Key Implications:**

- This list of research questions should inform and guide researchers and other stakeholders in research efforts on understanding the various aspects of respectful newborn care.
- Consensus-based definitions and standardized methodological research tools need to be developed to advance research efforts on respectful newborn care.

## BACKGROUND

Newborns are some of the most vulnerable members of society. Their dependency on others, inability to communicate verbally, and physical fragility, leave newborns potentially susceptible to disrespect and abuse. Studies have found that adverse experiences during the neonatal period, including pain^1^ and toxic stress,^2^ can have significant effects on physical health, mental wellbeing, and cognitive development later in life.^3^ At the same time, the receipt of evidence-based care practices, including pain management, breastfeeding, and gentle care, has been shown to reduce the risk of these adverse outcomes.^4^ With over 75% of newborns worldwide now delivered in health facilities,^5^ the focus on the provision of quality care for newborns and their families in health facilities is paramount. Quality of care for newborns includes not just standards relating to the provision of care, but also standards relating to the experience of care, including respect and dignity.^6^ As part of the respectful maternity care movement, recent efforts have been made to better understand and address the experiences of newborns in maternity care settings.

The “Respectful Maternity Care Charter: The Universal Rights of Childbearing Women”^7^ was first published in 2011 by the Global Respectful Maternity Care Council (GRMCC). Based on prevalent disrespect and abuse practices found in a 2010 landscape analysis, the Charter detailed 7 rights of women in the context of facility-based childbirth, grounded in various instruments of international law. These rights include the right to be free from harm and discrimination, the right to informed consent, privacy, and autonomy, and the right to the highest attainable standard of health, among others. Respectful maternity care received additional attention following the World Health Organization’s (WHO) 2014 statement on the “Prevention and Elimination of Disrespect and Abuse During Facility-Based Childbirth.”^8^ Significant work has been done since the 2014 statement to explore disrespect and abuse during facility-based childbirth, including manifestations and causes, to develop typology and operational definitions of mistreatment, and to advance interventions to reduce such practices and enhance respectful maternity care.^9^^,^^10^ Recent efforts to advance maternal and newborn quality of care have culminated in a set of comprehensive quality standards and associated measures and monitoring indicators. ^6^^,^^11^ As part of the agenda to go beyond survival to promote infant and child wellbeing, a framework was developed highlighting elements of nurturing care to improve early childhood development.^12^ In addition to clinical health, a nurturing care lens emphasizes good nutrition, safety, opportunities for learning, and responsive caregiving.

While the respectful maternity care movement has always recognized the importance of the mother-newborn dyad, until recently, it has focused primarily on the maternal perspective of disrespect and abuse during facility-based births. The care of the newborn was treated as part of maternity care, as the maternal and newborn experiences of care are interlinked and can often affect each other. However, with less attention given to the distinct experiences of newborns as independent beings, including potential disrespect and abuse, data collected on newborns’ experiences of care were lacking.

With less attention given to the distinct experiences of newborns as independent beings in the mother-newborn dyad, data collected on newborns’ experiences of care were lacking.

In 2017, a literature review focusing on newborns revealed incidences of mistreatment in most of the same categories identified as disrespect and abuse of women,^8^^,^^13^ but also called for additional research into the specific practices of newborn care.^14^ However, it is still unclear which elements of respectful care as related to newborns might be the most important, urgent, or feasible to address immediately.

In 2018, the GRMCC updated the Respectful Maternity Care Charter to include the rights of newborns during facility-based childbirth. Using the 2011 Charter’s framework, the GRMCC Newborn Working Group (NWG) expanded the scope of the Charter to 10 rights, covering the rights of newborns and articulating specific rights related to the protection of the mother-newborn dyad.^15^

We describe a prioritization exercise to identify global research questions using a modified Delphi process undertaken to catalyze and inform future research about the respectful treatment of newborns. This process resulted in a prioritized list of research questions in 3 categories.

## METHODS

The Delphi method has been established as a way of achieving consensus regarding research priorities among international experts.^16^ The method presents participants with a problem or question, and through a series of questionnaires, ideas are collected and recapitulated to create the next round of questionnaires. Participants rank the responses, which are recategorized and presented until a consensus is reached.

We modified the Delphi process (Figure) to include 2 rounds of consultation and feedback from experts with the aim of reaching a prioritized list but not necessarily a consensus. We aimed to identify potential research focus areas for understanding and addressing respectful care for newborns. Based on the findings of the 2017 literature review and expert discussions, we developed an open-ended questionnaire to identify key research categories. We asked participants, “What do you think are the most important research questions related to respectful maternal, newborn, and stillbirth care?” After consolidating and editing the results from Round 1, we created the Round 2 questionnaire, which asked participants to rank the preliminary list of research questions in 3 categories and generated a prioritized list of research questions.

**FIGURE:**
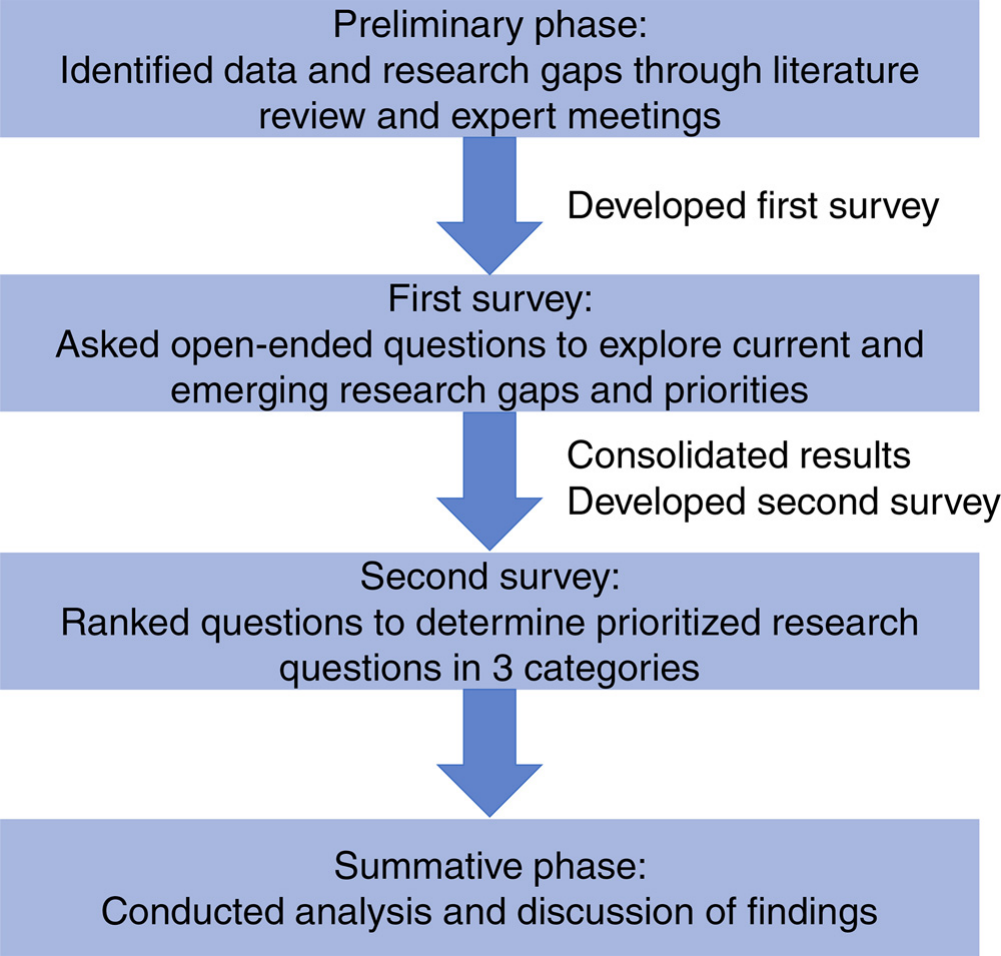
Modified Delphi Work Process Used to Identify a Prioritized List of Research Questions on Respectful Care for Newborns

Invited participants were researchers, practitioners, and advocates from around the world. Participants were recruited through the GRMCC, a network led by the White Ribbon Alliance, comprising more than 450 experts, representing over 150 international and local organizations from 45 countries. Members of the GRMCC include academics, researchers, health care practitioners, advocates, policy makers, and program administrators. Participants were encouraged to share the link to the survey with relevant members of their professional networks. Participation was anonymous unless the participant voluntarily provided an email address to receive future updates regarding the study; however, contact information was not linked to responses.

### Preliminary Phase

To define the scope of the discussion on the research priorities and prepare for the Delphi process, data regarding existing research gaps were collected using 2 data sources. First, key gaps in published research were identified through the literature review published in 2017. Second, during meetings held in May and June 2018, NWG members participated in discussions regarding gaps and priorities related to respectful care for newborns and were asked to suggest additional areas not appearing in the literature. Research gaps and priorities identified during the meetings were extracted from meeting minutes and summarized. These broad concepts served as the basis for the overarching survey questions presented in Round 1. For example, as some NWG members had shared that they had been involved in research or programs that focused mainly on respectful care of mothers but had some component of newborn care, we decided to ask separate questions about research focused primarily versus secondarily on newborns.

### Round 1: Open-Ended Survey

In June 2018, we sent members of the GRMCC a link to the first survey using Survey Monkey. Through open-ended questions, we asked participants to provide information: (1) about current research on respectful care for newborns, including objectives, geographic setting, and timeline; and (2) on what they perceived to be the most important concerns regarding respectful care for newborns, including particularly serious violations or particularly vulnerable groups. We invited participants to (1) suggest key research questions regarding respectful care for newborns, and (2) identify specific populations with the most urgent need for research on the issue (Box 1).

BOX 1 **Round 1 Survey Questions on Respectful Care for Newborns**

1. Are you currently involved in or aware of research that primarily focuses on respectful care for newborns/stillborn infants? If so, please provide details including objectives, country/location, timeline (ongoing/planned), donors, etc.
2. Are you currently involved in or aware of research that secondarily includes information about respectful care for newborns/stillborn infants? If so, please provide details including objectives, country/location, timeline (ongoing/planned), donors, etc.
3. When you think of respectful maternal, newborn, and stillbirth care, what do you think are the most important concerns (most prevalent issues or most serious rights violations)?
4. What do you think are the most important research questions related to respectful maternal, newborn, and stillbirth care?
5. Do you think there are priority populations or regions or vulnerable groups that need more urgent attention to respectful maternal, newborn, and stillbirth care?

We summarized the responses provided during the Round 1 qualitative survey and used them to compile the research questions for the Round 2 survey. We asked select NWG members to participate in a discussion about or provide written comments on the Round 2 survey instrument before dissemination.

### Consolidation Phase

Based on the consolidated responses to the Round 1 survey and discussions held in NWG meetings, we created an initial list of 32 research questions. Each question was analyzed to determine its subject area. Based on the subject areas that emerged, we grouped these questions into 3 categories. We sent these questions, along with 2 demographic questions, to select NWG members. These reviewers suggested further clarifying terminology, particularly regarding the definition of the term “respectful newborn care,” and adding several other potential research questions. Following the integration of the Round 1 survey results and the comments received from reviewers, we included a final list of 41 research questions in 3 categories in the Round 2 survey. The 3 categories were: descriptive, implementation, and measurement. In addition, we included several demographic and background questions regarding the experiences of survey participants with newborn care in the Round 2 survey. To ensure a level of consistency across respondents’ conceptualizations, we drafted and provided a definition of respectful care for newborns (Box 2).

BOX 2 **Definition of “Respectful Care for Newborns” Provided to Survey Participants**

The Respectful Maternity Care Council and Newborn Working Group place great importance in protecting the mother-baby dyad. However, for the sake of this survey only and because much work has already been conducted related to the care of women in childbirth, these research questions use the lens of how newborns and their families are treated. A consensus terminology has yet to be reached, but for this survey, we use the term “respectful care for newborns” to mean that the human rights of the newborn are protected, that newborns are treated with dignity and respect from the moment of birth, and that patient care is evidence-based and properly consented.

### Round 2 Ranked Survey

In February 2019, we created an online Round 2 survey using Survey Monkey. In March 2019, we invited all GRMCC members to participate in the survey, regardless of their participation in Round 1. We asked participants to share the invitation with relevant experts in their professional networks. To increase the likelihood of a geographical balance among participants, we sent individual survey participation requests to members located in frequently underrepresented geographic areas.

We asked participants to rank the 3 most important research questions in each of the 3 categories using a Likert scale, with “1” being the most important question in the category. After completing the ranking in all 3 categories, we invited participants to suggest additional research questions (“would you like to suggest a potential research question that is not included in the above categories?”). We used Microsoft Excel to calculate descriptive statistics for each category to identify the research questions that participants perceived as the most important. To determine the rank order in each category, we calculated frequencies and weighted mean scores. Comments and additional questions were analyzed and grouped by theme.

## RESULTS

### Preliminary Phase

A total of 21 experts participated in the 2 NWG member meetings held in May and June 2018, when preliminary research concepts were discussed to inform the survey development. Participants represented several governmental agencies, intergovernmental and nongovernmental organizations, and academic institutions. When discussing the evidence gap on respectful care for newborns, meeting participants noted: (1) a significant lack of evidence regarding the manifestations and prevalence of disrespect and abuse of newborns; (2) the need to develop indicators to measure disrespect and abuse of newborns; and (3) the need to conduct formative research to increase our understanding of perceptions of respectful care in various contexts and settings globally.

### Round 1: Open-Ended Survey

Round 1 survey aimed to compile an initial list of research questions related to respectful care for newborns based on experts’ opinions. In addition, the survey sought to identify current research projects related to respectful newborn care to see if some of the identified questions were already being studied, filling the evidence gaps. Round 1 resulted in 70 research questions from 25 respondents. We presented Round 1 survey results for discussion to the members of the NWG in September 2018. After review and analysis of the survey responses, several key areas of research and priority populations and settings emerged (Box 3). Finally, the survey results revealed very little extant research related to respectful care for newborns already underway as of 2019, and no studies primarily focused on newborns.

BOX 3 **Key Areas of Research, Priority Populations, and Settings That Emerged From Round 1 Survey Results Analysis**

Key areas of research

- Causes of disrespectful care for newborns and the effects of social and cultural norms among communities and health providers on these causes
- The promotion and integration of respectful care for newborns in health systems
- The effects and impact of respectful newborn care
- The appropriate measures and indicators for measuring respectful newborn care, both quantitatively and qualitatively

Priority populations and settings

- Socially and geographically marginalized groups
- Humanitarian crises settings
- Small and sick newborns (including those born preterm or requiring intensive medical care in neonatal intensive care units)
- Locations and communities with high rates of newborn mortality and stillbirth

The Round 1 survey results revealed very little extant research related to respectful newborn care already underway as of 2019.

### Round 2: Ranked Survey

#### Participants

A total of 52 participants completed the Round 2 survey. The majority of participants identified as researchers (53.2%) or as experts in program design, implementation, or evaluation (56.5%). More than half of the participants reported working primarily in low-income countries (66.0%). Participants’ characteristics are listed in Table 1.

**TABLE 1.** Characteristics of Participants in Round 2 Survey on Identifying Questions Related to Respectful Care for Newborns

	**No. (%)**
Gender^a^	
Female	36 (78.3)
Male	9 (19.6)
Other	1 (2.2)
Primary role related to newborn care^b^^,c^	
Research	25 (53.2)
Program design, implementation, or evaluation	27 (57.5)
Aid-work	5 (10.6)
Advocacy	12 (25.5)
Doctor	15 (31.9)
Midwife	6 (12.8)
Doula	1 (2.1)
Mother, parent, or other family	4 (8.5)
Other	6 (12.8)
Organization type	
Birth facility (including hospitals, clinics, and birth centers)	1 (2.1)
Government	5 (10.6)
Nongovernmental organization	23 (48.9)
University or research institute	16 (34.0)
Other	2 (4.3)
Main region of work^c^	
High-income countries	8 (17.0)
Middle-income countries	13 (27.7)
Low-income countries	31 (66.0)
Humanitarian settings	1 (2.1)

^a^ 46 participants answered the gender question.

^b^ Categories that received no answers are not included here.

^c^ Multiple answers allowed.

#### Research Questions

We calculated the percentage of participants who had ranked each question in the top 3 questions of the category. We then calculated the range and quartiles for each category of questions. Based on these ranges, importance was assigned as follows: high (Q1), intermediate (Q2 to Q3), and low (Q4). A weighted mean score was calculated for each question, using the importance assigned to it by participants, with 1 being the highest importance and 4 the lowest, assigned when the question was not ranked in the top 3 questions by a participant. The lowest mean represents the highest importance. The final ranking of questions in each category was determined based on the weighted mean score in each quartile category. Round 2 survey results for questions of high importance are presented in Table 2 and full results are in a Supplement.

**TABLE 2.** Ranking of Results of Round 2 Survey on Questions Related to Respectful Care for Newborns

	**Frequency in Top 3, No. (%)**	**Weighted Mean Score** ^a^	**Level of Importance (Top 3 in Category)**
Descriptive questions (N=52)			
What are the manifestations of disrespectful care or mistreatment of newborns that are observed in the context of facility based maternity care?	25 (48.1)	3.0	High (1)
How is respectful care of newborns defined by parents, by providers, and by the population in general?	19 (36.5)	3.1	High (2)
What are the perceptions and beliefs of health workers that affect the quality of care they provide to newborns?	15 (28.9)	3.4	High (3)
What is the prevalence of disrespect of newborns in health care facilities?	14 (26.9)	3.4	High (3)
Implementation questions (N=50)			
How can respectful care of newborns be promoted as the standard of care in a given health facility?	20 (40.0)	3.1	High (1)
How can health facility and management challenges be overcome to improve respectful care for newborns?	17 (34.0)	3.3	High (2)
What are the successful strategies for advocating for respectful care of newborns?	13 (26.0)	3.5	High (3)
Measurement questions (N=47)			
What are the measurable and appropriate quantitative metrics for respectful care of newborns?	35 (74.5)	2.1	High (1)
What is the impact of positive maternal prenatal and intra-partum experiences on birth outcomes?	17 (36.2)	3.3	High (2)
How can respectful care for newborns be measured qualitatively?	17 (36.2)	3.5	High (3)

^a^ The lowest mean represents the highest importance.

When respondents were asked to rank the top 3 questions in the Descriptive Questions category, “understanding what are the manifestations of disrespectful care and mistreatment of newborns” was considered the most important with a moderate level of agreement (ranked in the top 3 by 48.1% of participants). The next most important descriptive question was “how respectful care of newborns is defined” (36.5%). The last question to be categorized as important was “what perceptions and beliefs affect the quality care provided by health workers to newborns” (28.85%).

The second category, implementation questions, had greater variation in the prioritization of questions. In this category, the highest importance was assigned to “how to best promote respectful care of newborns as the standard of care” (40.0%). Participants also ranked highly the “identification of the challenges of health facilities and management in improving respectful care for newborns” (34.0%) and “assessing which strategies are most successful in advocating for respectful care of newborns” (26.0%).

The third category, measurement questions, had the highest level of agreement across the 3 categories, with the majority of participants (74.5%) ranking the question “what are the measurable and appropriate quantitative metrics for respectful care of newborns?” in the top 3 questions. The next highest-ranked measurement questions, which had the same level of agreement among participants (36.2%), were the “impact of positive maternal prenatal and intrapartum experiences on birth outcomes” and the “how can respectful care for newborns be measured qualitatively?”

Additional comments provided by participants focused primarily on issues related to the implementation of respectful care for newborns. Participants highlighted the need to explore how best to integrate respectful care into provider education and training and to articulate how culturally based practices can be best incorporated into such care. In addition, participants noted the need to research the cost-effectiveness of interventions. One participant emphasized the human rights aspect of respectful care, noting that such care should be provided regardless of whether it improves measurable health outcomes.

## DISCUSSION

We aimed to identify and rank a list of research questions about respectful care for newborns. To the best of our knowledge, this is the first attempt to explore what experts perceive to be the key global research questions for respectful care for newborns in a systematic way. To achieve this purpose, we invited experts representing a multitude of roles and experiences to participate in this study.

To the best of our knowledge, this is the first attempt to explore what experts perceive to be the key global research questions for respectful care for newborns in a systematic way.

The primary outcome of this modified Delphi process is a prioritized list of global research questions regarding respectful newborn care in 3 distinct categories of research: descriptive, implementation, and measurement. The final list of research questions ranked as highly important addresses issues vital to our understanding of respectful care for newborns.

The highest degree of agreement was in the measurement category, “what are the measurable and appropriate quantitative metrics for respectful care of newborns?” This indicates the need to understand the current prevalence of practices that could establish baseline figures which interventions or secular trends could be measured against. There was a wide range in the ranking of all other questions, with the next highest percentage of participants agreeing on a question being 48.1% (“what are the manifestations of disrespectful care or mistreatment of newborns that are observed in the context of facility-based maternity care?”), and the other top 3 questions’ agreement ranging from 26.0% to 36.0%. While the general level of agreement in this study was moderate, the questions ranked as highly important represent the need for fundamental data on the most basic topics related to respectful newborn care. The relatively wide scope of the top-ranked questions can also be explained by the perception of participants that basic, descriptive data are needed before advancing the discourse on respectful newborn care. The lack of shared definitions and standardized data regarding the causes, outcomes, and impact of how newborns are treated is further highlighted by the absence of clear measurement tools and guidelines. The outcomes of this study reflect both the lack of adequate data on respectful care for newborns and the need for experts engaged with newborn care for such data.

The discourse around respectful maternity care in recent years and the guidelines and interventions that stemmed from it have focused largely on the maternal aspects of the intra- and postpartum periods. This is due, at least in part, to the mother being the primary advocate for herself and her newborn and the one best situated to protect and report violations of their rights. This may also be due to the more obvious manifestations of disrespect and abuse of women during these sensitive periods and the ability of women to verbalize violations of their autonomy. However, despite evidence suggesting that newborns also experience violations of their rights, many of the nonclinical aspects of newborn care have received limited attention, and even some of the clinical aspects related to experience of care have been understudied. The findings of this study echo the development of the respectful maternity care movement. It took many years to develop, but experts and practitioners now have a shared language related to respectful maternity care and are interested in expanding it to include rights- and evidence-informed terms for respectful care for newborns within the context of respectful maternity care, as was done in the updated Respectful Maternity Care Charter.^6^ At the same time, as guidelines and interventions related to respectful care of women are being researched and implemented around the world, this study demonstrates the interest of experts in developing tools to advance respectful care for newborns, not simply as it relates to and affects their mothers, but as independent beings with their own rights.

Despite evidence suggesting that newborns also experience violations of their rights, many of the nonclinical aspects of newborn care have received limited attention.

This study demonstrates the interest of experts in developing tools to advance respectful care for newborns, not simply as it relates to and affects their mothers, but as independent beings with their own rights.

Integrating newborns into the respectful maternity care discourse provides additional protection to the mother-newborn dyad while ensuring that the unique needs of both mothers and newborns are addressed. However, while respectful care for mothers and newborns is interlinked, separate modules addressing the specific needs of newborns may be needed. Respectful care of newborns not only includes rights that cannot be separated from those of the mother, such as the support for breastfeeding, providing skin-to-skin contact, and minimizing separation, but also includes independent rights of newborns, including maintaining dignity, avoiding physical or verbal abuse, and being registered at birth. A key step in conceptualizing respectful newborn care and how to achieve it is understanding how such care can be measured. While experts in the field did not reach a clear consensus on other questions in this study, a vast majority agreed that identifying the appropriate quantitative metrics of respectful care for newborns is instrumental to advancing such care.

The advancements that have been made in the respectful care for newborns have focused thus far on small and sick newborns and stillborn infants. The particular circumstances of preterm or medically fragile infants have catalyzed queries from parents and family members about how to be best informed, involved, and supportive of their infants’ care. Small and sick newborns who are hospitalized for extended periods present with special needs and circumstances that are relevant in the study of mistreatment of newborns and respectful care for newborns. Access to quality health care is key and includes understanding how to best provide sensitive, gentle, and compassionate care, pain management, and palliative care. Implementing a nurturing care model, which includes many of these practices, improves health outcomes, and has long-term benefits for infant and child development.^12^ The unique needs of small and sick newborns have been recognized in the frameworks of several WHO guidelines, which also highlighted the need for additional data on newborns’ experiences of care and appropriate interventions.^11^

Although stillbirth-related questions were ranked by study participants as intermediate to low importance across all 3 categories, attention to respectful care has also been demanded by the families in the context of stillborn infants. Stillbirth research advocates have frequently noted that awareness among the general public is low and that the importance of effective bereavement care and related research may not be given much thought by those who have not experienced or been affected by a loss.^17^ Through consultative processes with families, providers, and researchers, principles for respectful bereavement care have been developed but with limited implementation.^18^ In addition to the need for respectful care to bereaved parents, further research is needed to advance dignified care for stillborn infants and those who do not survive the neonatal period, including offering the family options, which may include a funeral and registration of the birth and death.^19^

Preliminary research from secondary data shows that the care that many newborns receive fails to meet quality of care standards, both in terms of provision of care and experience of care,^4^ and that poor experiences of care can often constitute mistreatment.^20^ Some observed practices such as “non-gentle handling” may be particularly difficult to measure objectively, but others, such as being slapped or held upside down, are more easily identified as mistreatment. A recent observational study of more than 30,000 newborns in Nepal revealed that many failed to receive respectful care in health facilities, including receiving medical interventions without parental consent and rough handling.^21^ The high prevalence of newborns separated from their families has also been documented, although further work is needed to understand how much, if any, is driven by medical need and how often families understand and consent to such care.^22^

The coronavirus disease (COVID-19) pandemic and the response to it within health systems and facilities have further exposed not only harmful practices but also the disparities in experiences of care.^23^ Early on in the pandemic, significant shifts in maternity care delivery were observed across the world, including reverting to care practices that have long been deemed as failing to meet quality of care standards.^24^ In many places, newborns were separated from their mothers and were prevented from breastfeeding.^25^ In response, the WHO provided guidance regarding newborn care in health facilities during the pandemic, highlighting the importance of providing respectful care for newborns even during challenging times.^26^

As the experiences of the COVID-19 pandemic and its effects on respectful care for newborns are still being studied, further research should go beyond and explore how public health emergencies and other potentially exacerbating circumstances affect newborn care and what standardized tools can be used to document practices and their changes and impact over time.

These ranked research questions should inform further research efforts on respectful newborn care, particularly the development of consensus-based definitions and standardized methodological research tools. Parallel to research efforts, policy makers and program implementers can begin improving care by including parents and families in discussions about preferences and how to develop supportive policies. For example, a recent WHO roadmap for improving newborn care in health facilities refers to the need to integrate respectful and family-centered care into health professionals’ training and experience and quality of care standards.^27^ In addition, the roadmap calls for ensuring there are sufficient numbers of qualified and skilled health care workers.

Parallel to research efforts, policy makers and program implementers can begin improving care by including parents and families in discussions about preferences and how to develop supportive policies.

Holistic research agendas should strive to understand the challenges faced by health care workers and the support they need to provide high-quality and respectful care to their patients. Pre-service and in-service training for health care workers can be strengthened by adding components of respectful and inclusive care. Health workers should also be seen as advocates for improved care of patients, and facility policies and infrastructure should be adjusted where possible to facilitate more respectful care, including allowing for privacy, rooming-in, and as much parental and family visitation as possible. These efforts can be further strengthened through implementation research on how to achieve respectful care for newborns through continuous improvement of various interventions.

Special attention should be paid to the needs of vulnerable groups, including small and sick newborns. In addition, better alignment and coordination are needed with other movements striving to improve newborn health care and outcomes, including family-centered care and the Every Newborn Action Plan.^28^

### Strengths and Limitations

The strengths of this study include the participants’ representation of many of the leading entities involved in research, policy, and programming of newborn care globally. We likely increased participation by not requiring respondents of the second survey to have participated in the first survey, but this may have limited the options of respondents who participated only in the second survey. While we recognize that the study sample was small and that the study participants cannot be considered representatives of all stakeholders relevant to newborn care, we believe that the relative diversity and level of expertise of the participants, together with the fundamental nature of this research topic, represent priorities that are shared across various settings. While not comprehensive, many group members had links to other networks and were able to give information about ongoing research or lack thereof beyond the direct membership of the GRMCC. Further, as the field of respectful maternity care research is relatively new and inclusion of newborns in the research agenda even newer, we believe the sample size represents a sizeable proportion of the professionals involved in this field at the global level. As initiatives expand, more contextualized research will be needed across settings, and regional and country-level experts will be needed to design appropriate programs and strategies to promote respectful care given the available staff, space, and resources.^29^

This study provided, for the first time, a working definition of respectful care for newborns. While more research is needed to better understand the various aspects of respectful care for newborns, the definition provided in this study can be used as a shared starting point for further work in this emerging field. One limitation is that this survey was provided in English; further work will be required in the future to globalize these concepts across settings and languages.

The Delphi method proved useful in both developing a list of research questions by soliciting multiple opinions and narrowing the list to the most important questions based on the most pressing needs as identified by experts. The ability to present a substantial list of questions to experts but allowing for additional suggestions at each stage aided in reducing the concern that key research questions have been overlooked. The lack of agreement on the priorities for the majority of questions is a clear limitation of this study, as it may not have been possible to ever reach consensus. However, it also represents the emerging nature of the issue of respectful care for newborns and the need to create a shared language and a research agenda to advance discourse and action on this issue.

## CONCLUSION

This study developed, for the first time, a ranked list of research questions focusing exclusively on respectful care for newborns. By mobilizing experts in the field to identify and prioritize key research questions, the study highlighted the absence of agreed-upon terminology and tools needed to advance both theoretical and practical research efforts related to respectful care for newborns. Every newborn should have their rights protected regardless of the circumstances of their birth. The list of research priorities generated in this study should guide researchers and other stakeholders in developing further research projects, instruments, and trajectories aimed at advancing respectful care for newborns.

## Supplementary Material

GHSP-21-00292-Supplement.pdf

## Notes

**Peer Reviewed**

**First published online:** February 3, 2022.
