# Supplementary material for: Global Research Priorities for Understanding and Improving Respectful Care for Newborns: A Modified Delphi Study
Source: Glob Health Sci Pract. 2022 Feb 28;10(1):e2100292. doi: 10.9745/GHSP-D-21-00292 (PMC8885351; doi:10.9745/GHSP-D-21-00292)
Supplement: GHSP-21-00292-Supplement.pdf [file GHSP-21-00292-Supplement.pdf]

Supplement to: Palgi-Hacker H, Ateva E, Jolivet R, et al. Global research priorities for understanding and improving respectful care for newborns: a modified delphi study. *Glob Health Sci Pract*. 2022;10(1). https://doi.org/10.9745/GHSP-D-21-00292

Supplement. Ranking of Full Results of Round 2 Survey on Questions Related to Respectful Newborn Care

Descriptive Questions (N=52)	Frequency in Top 3, No. (%)	Weighted Mean Score[a]	Level of Importance (Top 3 in Category)
What are the manifestations of disrespectful care or mistreatment of newborns that are observed in the context of facility based maternity care?	25 (48.1)	2.96	High (1)
How is respectful care of newborns defined by parents, by providers, and by the population in general?	19 (36.5)	3.10	High (2)
What are the perceptions and beliefs of health workers that affect the quality of care they provide to newborns?	15 (28.9)	3.40	High (3)
What is the prevalence of disrespect of newborns in health care facilities?	14 (26.9)	3.42	High
What are the main causes of unnecessary separation between the mother and newborn in health facilities?	13 (25.0)	3.44	Intermediate
What challenges do health facilities (including management, workers and infrastructure) experience, that limit or inhibit respectful care of newborns?	12 (23.1)	3.50	Intermediate
What types of disrespect of newborns are of most concern to mothers and families?	8 (15.4)	3.71	Intermediate
What beliefs and norms affect the way small and sick newborns are treated?	7 (13.5)	3.71	Intermediate
What are the social norms among communities and family members that influence how newborns are treated?	9 (17.3)	3.75	Intermediate
What challenges do health facilities (including management, workers and infrastructure) experience, that limit or inhibit respectful care of small and sick newborns?	6 (11.5)	3.77	Intermediate
What beliefs and norms affect the way stillbirths and stillborn infants are treated?	7 (13.5)	3.77	Intermediate
How do health facility workers explain or justify their actions, and do they perceive any of their actions as disrespectful of newborns?	7 (13.5)	3.85	Intermediate
What do health facility workers believe is necessary to improve respectful care of newborns in their facility?	6 (11.5)	3.85	Intermediate
Which aspects of treatment of newborns are influenced by motivation issues among the health facility workers (stemming from overwork, crowding, lack of pay, professional respect, etc.)?	3 (5.8)	3.88	Low

Supplement to: Palgi-Hacker H, Ateva E, Jolivet R, et al. Global research priorities for understanding and improving respectful care for newborns: a modified delphi study. *Glob Health Sci Pract*. 2022;10(1). https://doi.org/10.9745/GHSP-D-21-00292

**Descriptive Questions (N=52)**	**Frequency in Top 3, No. (%)**	**Weighted Mean Score[a]**	**Level of Importance (Top 3 in Category)**
Are some sub-groups of newborns more likely to experience disrespectful care than others and, if so, which groups are most vulnerable?	3 (5.8)	3.92	Low
Besides social norms and health system constraints, what other drivers contribute to poor treatment of newborns or stillborn infants?	2 (3.9)	3.96	Low
**Implementation Questions (N=50)**			
How can respectful care of newborns be promoted as the standard of care in a given health facility?	20 (40.0)	3.08	High (1)
How can health facility and management challenges be overcome to improve respectful care for newborns?	17 (34.0)	3.28	High (2)
What are the successful strategies for advocating for respectful care of newborns?	13 (26.0)	3.48	High (3)
What are the behaviors of health care workers that are the most difficult to change, and how can they be addressed?	12 (24.0)	3.48	Intermediate
How can effective models of care that allow for minimal separation between mother and newborn during their stay in the health facility be implemented in health facilities?	11 (22.0)	3.50	Intermediate
How can integrated care for both the mother and newborn best be provided in low resource settings, especially for high-risk cases?	12 (24.0)	3.52	Intermediate
How can effective models of care for the small and sick newborn that combine concepts of respect, compassion, developmentally supportive care, and family centered care be best implemented, especially in low income settings?	11 (22.0)	3.56	Intermediate
How can respectful care of newborns care be promoted as the standard of care at a district or national level?	12 (24.0)	3.58	Intermediate
What motivates governments to invest resources in respectful care for newborns?	8 (16.0)	3.64	Intermediate
How can effective interventions to address social norms be implemented to improve care for stillborn infants and their families?	10 (20.0)	3.66	Intermediate
How can effective models of care for that combine concepts of respect, compassion, developmentally supportive care, and family centered care be best adapted to humanitarian settings?	8 (16.0)	3.74	Intermediate
How can power imbalances between health care providers and patients be addressed to improve care for newborns?	7 (14.0)	3.76	Low

Supplement to: Palgi-Hacker H, Ateva E, Jolivet R, et al. Global research priorities for understanding and improving respectful care for newborns: a modified delphi study. *Glob Health Sci Pract*. 2022;10(1). https://doi.org/10.9745/GHSP-D-21-00292

**Descriptive Questions (N=52)**	**Frequency in Top 3, No. (%)**	**Weighted Mean Score[a]**	**Level of Importance (Top 3 in Category)**
How can health facility and management challenges be overcome to improve care for newborns and their families?	5 (10.0)	3.86	Low
What logistical and administrative barriers need to be addressed to improve respectful care of stillborn infants?	6 (8.0)	3.92	Low
**Measurement Questions (N=47)**			
What are the measurable and appropriate quantitative metrics for respectful care of newborns?	35 (74.5)	2.11	High (1)
What is the impact of positive maternal prenatal and intra-partum experiences on birth outcomes?	17 (36.2)	3.28	High (2)
How can respectful care for newborns be measured qualitatively?	17 (36.2)	3.45	High (3)
What is the impact of respectful care of newborns on the health of newborns?	14 (29.8)	3.34	Intermediate
How can unnecessary separation of the newborn from the mother be measured in quantitative surveys?	9 (19.2)	3.45	Intermediate
How does disrespectful care of small and sick newborns affect their survival and ability to thrive?	13 (27.7)	3.49	Intermediate
What are the long-term impacts of respectful or disrespectful care of newborns on infant neurodevelopment and early childhood development?	12 (25.5)	3.55	Intermediate
What is the impact of respectful care of newborns on future health care utilization for the newborn/child?	7 (14.9)	3.70	Intermediate
Which interventions to improve care for stillborn infants and their families have the greatest impact on patient satisfaction with care?	6 (12.8)	3.79	Low
What effect does respectful care during stillbirth and of the stillborn infant have on maternal health?	7 (14.9)	3.81	Intermediate
How do we measure denial or threat of denial of care to newborns?	1 (2.1)	3.98	Low

[a] The lowest mean represents the highest importance.
